# Efficient homology‐based annotation of transposable elements using minimizers

**DOI:** 10.1002/aps3.11520

**Published:** 2023-05-11

**Authors:** Laura Natalia Gonzalez‐García, Daniela Lozano‐Arce, Juan Pablo Londoño, Romain Guyot, Jorge Duitama

**Affiliations:** ^1^ Systems and Computing Engineering Department Universidad de los Andes Bogotá Colombia; ^2^ UMR DIADE, Institut de Recherche pour le Développement Université de Montpellier, CIRAD 34394 Montpellier France; ^3^ Department of Biological Sciences Universidad de los Andes Bogotá Colombia

**Keywords:** bioinformatics, genomics, software, transposable elements

## Abstract

**Premise:**

Transposable elements (TEs) make up more than half of the genomes of complex plant species and can modulate the expression of neighboring genes, producing significant variability of agronomically relevant traits. The availability of long‐read sequencing technologies allows the building of genome assemblies for plant species with large and complex genomes. Unfortunately, TE annotation currently represents a bottleneck in the annotation of genome assemblies.

**Methods and Results:**

We present a new functionality of the Next‐Generation Sequencing Experience Platform (NGSEP) to perform efficient homology‐based TE annotation. Sequences in a reference library are treated as long reads and mapped to an input genome assembly. A hierarchical annotation is then assigned by homology using the annotation of the reference library. We tested the performance of our algorithm on genome assemblies of different plant species, including *Arabidopsis thaliana*, *Oryza sativa, Coffea humblotiana*, and *Triticum aestivum* (bread wheat). Our algorithm outperforms traditional homology‐based annotation tools in speed by a factor of three to >20, reducing the annotation time of the *T. aestivum* genome from months to hours, and recovering up to 80% of TEs annotated with RepeatMasker with a precision of up to 0.95.

**Conclusions:**

NGSEP allows rapid analysis of TEs, especially in very large and TE‐rich plant genomes.

The size of plant genomes varies enormously, by a factor of ~3000 (65 Mbp to 148.9 Gbp), due to polyploidy events and the accumulation of transposable elements (TEs) (Michael, 2014; Kress et al., 2022). These TEs represent the major part of the genome sequence of plants (Tenaillon et al., 2010). Such an accumulation of TEs has an important impact on the structure and composition of chromosomes, as well as on the function, expression, and regulation of genes, playing an important role in the adaptive evolution of species (Lisch, 2013). Different studies have shown that the mobility of TEs can have an important positive impact on traits of agronomic interest for crop species. TEs have been related to fruit pigmentation in apples (*Malus domestica* (Suckow) Borkh.) (Zhang et al., 2019) and the nectarine phenotype in peaches (*Prunus persica* (L.) Batsch var. *nucipersica* Dippel) (Vendramin et al., 2014). In general, TEs contribute to the diversity of crops, such as rice (*Oryza sativa* L.) and tomato (*Solanum lycopersicum* L.) (Carpentier et al., 2019; Domínguez et al., 2020); they also participate in the evolution of biosynthetic gene clusters that produce secondary metabolites related to environmental adaptation (Bharadwaj et al., 2021), and they can be related to crop disease resistance (Zervudacki et al., 2018). As expected, TE dynamics can also have a negative impact on agronomic traits, as illustrated by the mantled mutation in clonally propagated *Elaeis guineensis* Jacq. (oil palm) (Ong‐Abdullah et al., 2015). The identification and annotation of TEs is a required step in the analysis of plant genome assemblies. Nevertheless, these tasks are challenging for very large and complex genomes due to the collapse of their copies during the assembly process, their structural polymorphisms, high divergence, high copy number, and the ability of TEs to move to different areas of the genome (Bourque et al., 2018).

Different algorithms have been developed to identify and annotate TEs. TE annotation methods have been classified according to the information used to identify regions with potential TEs as de novo methods, signature‐based methods, structure‐based methods, learning‐based methods, homology‐based methods (also called library‐based methods), and consensus‐based methods (consensus of two or more of the previously listed methods) (Loureiro et al., 2013; Girgis, 2015). In particular, the homology‐based methods usually detect and annotate TEs using a curated reference library of sequences from a closely related species. The identification is achieved using alignment algorithms, with BLAST and BLAST‐like tools being the most commonly implemented. This approach is used in tools such as RepeatMasker (Smit et al., 1996), Censor (Jurka et al., 1996), or BLASTER/MATCHER (Quesneville et al., 2003). However, these tools rely on classic alignment algorithms, which are not computationally efficient compared to new alignment methodologies, especially when dealing with very large reference genomes (Alser et al., 2021). Moreover, the heuristic algorithm implemented in BLAST reduces the sensitivity to find distant matches. Tools based on hidden Markov models, such as the Dfam pipeline, have been developed to identify distant matches, but at an additional computation cost (Storer et al., 2021).

Although sequence alignment is a classic research topic in bioinformatics, it still represents a challenge due to the current availability of long‐read sequencing technologies and genome assemblies for an increasing number of species. The vast amount of data generated by these technologies has motivated the development of novel algorithms to improve computational efficiency over the traditional algorithms to perform pairwise alignments, such as the Smith–Waterman algorithm (Smith and Waterman, 1981). One approach is to reduce the search space by storing subsequences, called minimizers, which can represent the entire sequence (Roberts et al., 2004). In particular, the use of minimizers has been implemented as a base for efficient alignment of error‐prone long DNA sequencing reads (Li, 2018), for identification of read overlaps in de novo genome assembly (Ruan and Li, 2019), for genome assembly alignment, and for building pan‐genome graphs (Li et al., 2020). In these applications, a numerical function termed the hashing function is applied to all the overlapping *k*‐mers that can be extracted from a window of length *w* from the sequence. For each window, a minimizer is defined as a *k*‐mer that generates the minimum value of the hashing function (Li, 2016). Given an appropriate hash function, the minimizers provide an unbiased subsampling of the complete catalog of *k*‐mers. Comparing minimizer matches becomes an efficient alternative method to identify homologous DNA sequences, as compared to the calculation of matches over the complete set of *k*‐mers.

Here, we present the development of a minimizer‐based approach to implement an “alignment free” TE annotation tool for genome sequences using a reference library. Benchmark experiments on *Arabidopsis thaliana* (L.) Heynh. (thale cress), *Oryza sativa* L. (cultivated rice), *Coffea humblotiana* Baill. (wild coffee), and *Triticum aestivum* L. (bread wheat) show that our algorithm represents an improvement in efficiency in the use of computational resources, while achieving good sensitivity and precision. This algorithm is implemented as a functionality of the Next‐Generation Sequencing Experience Platform (NGSEP) (Tello et al., 2023), allowing a tight integration with the genome assembly and further annotation pipelines.

## METHODS AND RESULTS

### Algorithm based on minimizers for identification of genomic regions with transposable elements

**Figure 1:**
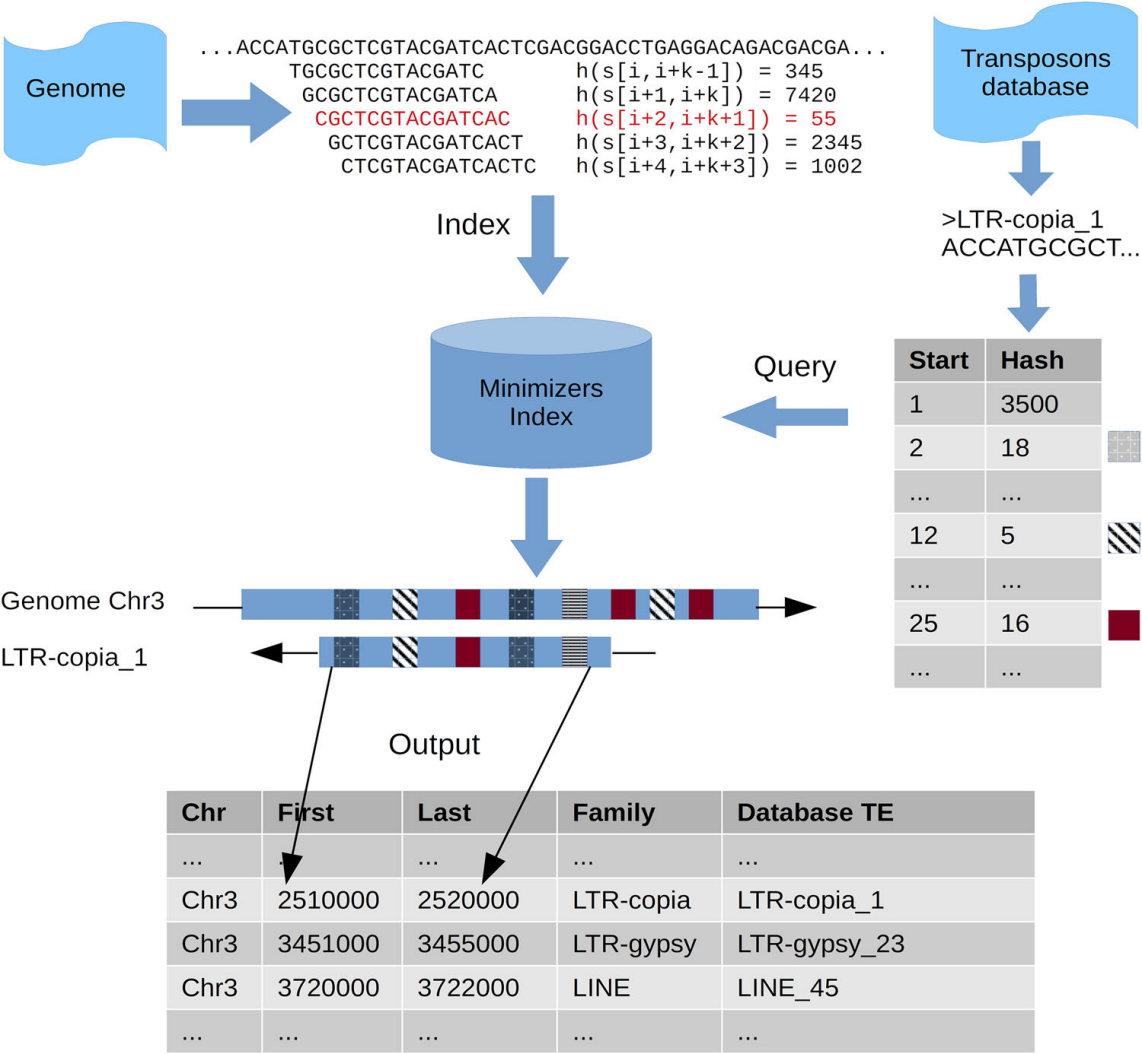
Minimizer‐based search of transposable elements (TEs) in an assembled genome. The genome is indexed using minimizers. *k*‐mers of each TE in the curated library are calculated and searched in the minimizers index. Dynamic programming is used to cluster hits consistent with a single TE copy. The start of the first clustered *k*‐mer hit and the end of the last clustered *k*‐mer hit are used as boundaries for the identification of the element.

We developed a new algorithm to perform computationally efficient identification of TEs in a genome assembly (Figure 1). The process receives the genome assembly to annotate in FASTA format and a FASTA database of curated TEs as input. These elements can initially be extracted from a closely related species or can be inferred from a de novo analysis of the genome, running tools such as the long terminal repeat (LTR) de novo annotator Inpactor2 (Orozco‐Arias et al., 2022). Curated reference databases, such as the complete TEs database Repbase (Bao et al., 2015) or the LTR retroelements database InpactorDB (Orozco‐Arias et al., 2021), can also be used as TE libraries. Input libraries and genomes can contain International Union of Pure and Applied Chemistry characters.

The fundamental principle of the algorithm is to consider curated TEs as noisy long reads. Hence, minimizers can be applied to map instances of these TEs onto the genome, following the same core ideas implemented in Minimap2 (Li, 2018). Our algorithm builds a minimizers table for the reference genome with a fixed *k*‐mer length of 15 bp and a window length of 20 bp. Briefly, for each overlapping window of length 20 bp, all overlapping *k*‐mers (substrings) with a length of 15 bp are extracted. Then, a numerical hashing function is applied to each *k*‐mer. This function can be viewed as a pseudo‐random number generator, which can be used to sample consistent *k*‐mers in homologous sequences, regardless of minor presence‐absence variation between the sequences (see Li, 2016). For each window, the *k*‐mer with the minimum value of the hashing function is selected, and the *k*‐mer sequence and location are stored in the minimizers table. As neighbor windows can be represented by the same minimizer, such a minimizer and its location are stored only once in the minimizers table. For each input TE, *k*‐mers are extracted and matched to the reference genome. Similar to Minimap2, dynamic programming is used to cluster *k*‐mer hits supporting the same TE copy, with up to 10,000 hits retained for each input TE. To make the process computationally efficient, a complete alignment of the input TE is not performed. Instead, the first and the last reference positions of each cluster of *k*‐mer hits are retained. These positions then become the boundaries of the TE match. Matches identified by each input TE are collected, and overlapping matches are merged into one match.

Because the input TEs from the curated library cannot cover the complete representation of the diversity across all TE families of the analyzed genome, a second mapping step is necessary. This second step is carried out using the curated library merged with the TE annotations obtained in the first round as the input library. The objective of this second round of searches is to increase the sensitivity at the cost of increased runtime. The number of iterations can be set by the user (two iterations by default).

TE annotations are provided as text files with four columns: sequence name (chromosome), first reference position, last reference position, and the family name of the curated TE that was mapped to generate the annotation. We also developed a feature to mask a genome assembly given a text file with at least the first three columns. Users can select either soft masking, which converts the annotated regions to lowercase, or hard masking, which places ‘N’ characters in the annotated regions.

### Implementation details

We implemented the algorithm described above as part of the NGSEP (Tello et al., 2023). This software is coded in Java following object‐oriented software design patterns. We reused several classes of the NGSEP infrastructure implemented for different purposes, including the management of genome assemblies and genomic regions, the minimizers table, and implementation of the algorithm to align long reads to reference genomes. The functionalities to annotate TEs and to mask genomes are available, both in the command line interface (commands TransposonsFinder and GenomeAssemblyMask, respectively) and in the graphical interface, and were released in version 4.3.1. Stable versions of NGSEP are available in SourceForge (https://ngsep.sf.net), and continuous development is available on GitHub (https://github.com/NGSEP). Hereafter, we will refer to the functionality presented in this work as NGSEP‐TF.

### Benchmark data sets and software tools

To perform benchmark experiments of the algorithm presented above, publicly available genomes were retrieved, as well as curated TE libraries. (See the Data Availability Statement for databases and accession numbers.) *Arabidopsis thaliana* TAIR10.1, which has less than 15% of TEs identified, was included as a compact genome. Medium genomes in terms of TE content included the *O. sativa* ‘Nipponbare’ genome assembly (Kawahara et al., 2013) and *C. humblotiana* (Raharimalala et al., 2021). These two genomes contain between 30% and 50% of TE content. Finally, as a complex data set (~90% TEs), the hexaploid genome of *T. aestivum* was included (Zhu et al., 2021).

We compared the performance of the algorithm described in this work with the algorithm implemented in RepeatMasker v4.1.2‐p1 (Smit et al., 1996). Comparisons were carried out using LTR and non‐LTR TE libraries. Both NGSEP‐TF and RepeatMasker were executed using default parameters; however, for RepeatMasker results were filtered to remove low‐complexity regions and simple repeats. In both cases, TEs shorter than 200 bp were removed. All processes were run in a Xeon Gold computing node (Intel, Hillsboro, Oregon, USA) with 42 threads and 565 GB of RAM (jobs restricted to 32 threads and 64 GB of RAM); the *T. aestivum* genome required approximately 250 GB of RAM to run each chromosome separately.

To compare the identified TEs from both tools, we implemented an in‐house script based on the NGSEP code and made it available with the NGSEP distribution (class ngsep.genome.GenomicRegionSetsComparator). This script takes as input two genome annotation data sets and calculates for each data set the number and percentage of base pairs that are annotated in the other annotation data set. We used this script to calculate the fraction of base pairs annotated by NGSEP‐TF that were also annotated by RepeatMasker (precision) and the fraction of base pairs annotated by RepeatMasker that were also annotated by NGSEP‐TF (sensitivity).

### Comparison between the NGSEP‐TF and RepeatMasker with reference LTR retrotransposons

**Figure 2:**
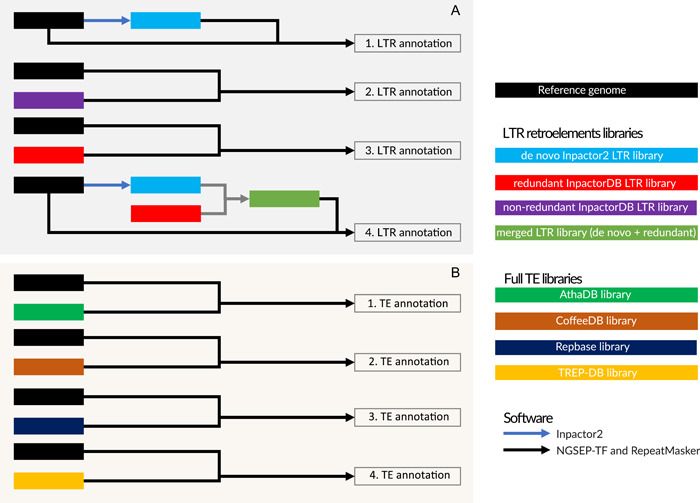
Benchmarking experiments (A) to annotate LTR retrotransposons and (B) to annotate full TEs. In (A), (1) represents the LTR annotation using de novo Inpactor2 libraries, (2) represents the LTR annotation using the merged library (de novo Inpactor2 library + non‐redundant InpactorDB library), (3) represents the LTR annotation using the redundant InpactorDB library, and (4) represents the LTR annotation using the non‐redundant InpactorDB library. In (B), (1) represents the TE annotation using the curated *Arabidopsis thaliana* library (AthaDB); (2) represents the TE annotation using the curated *Coffea humblotiana* library (CoffeeDB), (3) represents the TE annotation using the curated Repbase v20.05 database (Repbase), and (4) represents the TE annotation using the curated TREP database (TREP‐DB). The same pipelines were applied to each data set (*Arabidopsis thaliana, Coffea humblotiana*, and *Oryza sativa*).

To assess the accuracy and efficiency of NGSEP‐TF for identification of LTR retrotransposons, we built de novo LTR retrotransposon libraries and downloaded curated LTR retrotransposon libraries (Figure 2A). We focused first on LTR retrotransposons because they represent the vast majority of TEs in plant genomes. To obtain de novo LTR retrotransposon libraries for each species, we ran the software Inpactor2 (Orozco‐Arias et al., 2022) using the reference genomes as input. These de novo libraries did not follow a manual curation step. The *A. thaliana* Inpactor2 library contained 109 LTR retroelements, the *O. sativa* 1088 TEs, and the *C. humblotiana* 876 TEs. These TEs are classified into superfamilies and lineages. We executed both the NGSEP‐TF and RepeatMasker on each genome assembly, using its corresponding de novo library. Both tools annotated less than 10% of the *A. thaliana* genome assembly with LTR retrotransposons, having an intersection of 4.8 Mbp as annotated TEs. In *O. sativa* and *C. humblotiana*, which were used as medium complexity genomes, both tools annotated between 21% and 27% of the genome assemblies with LTR retrotransposons.

**Figure 3:**
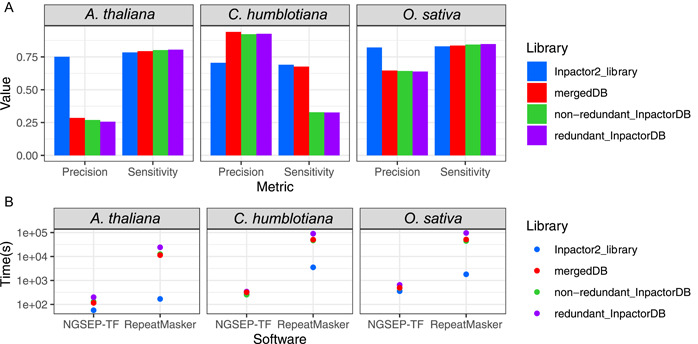
Comparison of NGSEP‐TF and RepeatMasker LTR annotation using LTR libraries for *Arabidopsis thaliana, Coffea humblotiana*, and *Oryza sativa*, showing (A) the precision and sensitivity of NGSEP‐TF using RepeatMasker as a reference, and (B) the time consumption of both tools using 32 cores and 64 GB RAM. For each species, the merged library corresponds to the de novo Inpactor2 library plus the non‐redundant InpactorDB library.

Unfortunately, in this case, it is very difficult to build an orthogonal gold‐standard data set that can be used to perform a formal benchmark experiment between the tools, because TEs of nearly all current genome assemblies have been annotated using RepeatMasker. Hence, instead of trying to compare the accuracy of the two software tools, we took annotations of RepeatMasker as the gold standard to validate the accuracy of NGSEP‐TF annotations as their similarity to RepeatMasker annotations. Figure 3A shows the overall precision and sensitivity obtained for our algorithm following the described approach. The precision of the NGSEP‐TF annotations ranged between 0.70 and 0.75, whereas the sensitivity ranged from 0.69 to 0.83. The best values were observed in the annotation of the *O. sativa* genome (0.82 precision and 0.83 sensitivity), and the worst values were observed in the *C. humblotiana* genome (0.70 precision and 0.69 sensitivity). We evaluated the number of iterations in the NGSEP‐TF algorithm by repeating the *A. thaliana* and *O. sativa* experiments, running one to three iterations of NGSEP‐TF. In both cases, the sensitivity increased with the number of iterations (0.65 to 0.83); however, the precision decreased (0.96 to 0.47) (Appendix S1). The best F‐score was obtained running one iteration for *A. thaliana* (0.78) and two iterations for *O. sativa* (0.82). In addition, we investigated the performance of NGSEP‐TF using two LTR public libraries: the redundant and the non‐redundant manually curated LTR retrotransposon libraries from InpactorDB (Orozco‐Arias et al., 2021). These libraries contain LTR retroelements belonging to all lineages, and they were used as the training libraries of the de novo annotator Inpactor2. We also merged the redundant InpactorDB library with the de novo libraries for each species (represented in Figure 3 as “mergedDB”). For *A. thaliana* and *O. sativa* annotations, the sensitivity improved while the precision decreased. In contrast, the opposite trend was observed in the annotations of the *C. humblotiana* genome.

Unlike accuracy, computational efficiency can be compared between the tools without the need of a gold standard. Hence, we compared the computational runtimes needed by NGSEP‐TF and RepeatMasker to obtain annotations of TEs. As shown in Figure 3B, NGSEP‐TF required between one and two orders of magnitude less runtime than RepeatMasker, passing from hours to minutes or seconds. On average, NGSEP‐TF was capable of annotating the genomes in 5 min, whereas RepeatMasker took 10 h. Detailed times are shown in Appendix S2.

**Figure 4:**
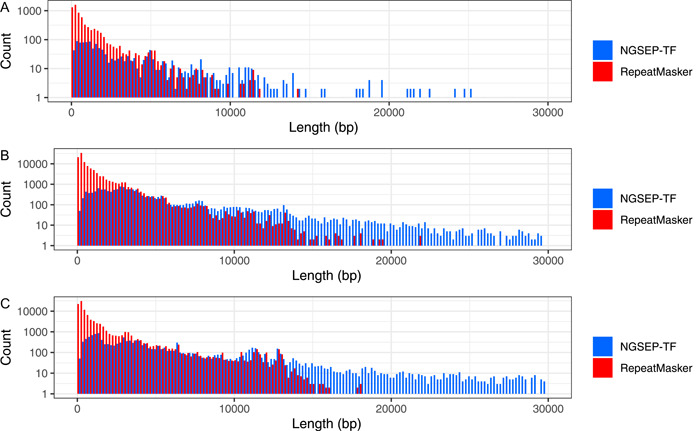
Comparison of LTR retrotransposon length distribution between NGSEP‐TF and RepeatMasker for (A) *Arabidopsis thaliana*, (B) *Coffea humblotiana*, and (C) *Oryza sativa*. Each bar represents a step of 200 bp. On the *y*‐axis, the count represents the number of TEs with the specified length.

As a first approach to understanding the differences among tools, the distribution of lengths of annotated TEs was analyzed. Complete LTR retrotransposons in plants commonly have lengths from 4000 to 20,000 bp, according to their family (Llorens et al., 2011). RepeatMasker annotated shorter elements (<1000 bp), which are either incomplete LTR retroelements or misclassified regions (Figure 4). The NGSEP‐TF algorithm annotates one order of magnitude fewer of these small regions as LTR retrotransposons. Conversely, the NGSEP‐TF annotations include large regions. Some of these cases probably correspond to colocalized TE insertion events that the current algorithm merges into a single annotation (Appendix S3).

Finally, to evaluate the capacity of NGSEP‐TF and RepeatMasker to call intact LTRs, TE annotations obtained with both tools using the de novo Inpactor2 libraries were compared against the genomic location of the TEs included in the input libraries themselves. Using NGSEP‐TF, the sensitivity was 0.9986 for *A. thaliana*, 0.9989 for *O. sativa*, and 0.9987 for *C. humblotiana*, while using RepeatMasker, sensitivity values were 0.997 for *A. thaliana*, 0.9995 for *O. sativa*, and 0.7463 for *C. humblotiana*. For TE coverage, NGSEP‐TF annotations covered, on average, 99.85% of the length of the elements in the library, whereas RepeatMasker annotations covered, on average, 91.15% of the length of the elements. Appendix S4 shows the distribution of the coverage percentage for both tools using the length of the elements in the library and the length of the elements in the annotations as the size references.

### Comparison between NGSEP‐TF and RepeatMasker with a complete representation of TEs

We also assessed the accuracy and efficiency of NGSEP‐TF to identify non‐LTR transposons. Because Inpactor2 only predicts LTRs, publicly available reference libraries were used in this case to generate these annotations. (See the Data Availability Statement for databases and accession numbers.) Curated libraries included the *C. humblotiana* curated TE library (Sol Genomics Network, 2023), the *A. thaliana* library (Plant Bioinformatics Facility, 2023), the updated non‐redundant nucleotide TREP database (Schlagenhauf and Wicker, 2016), and the Repbase library v.20.05 (Bao et al., 2015) (Figure 2B). The *A. thaliana* library contains 326 TEs. The *C. humblotiana* library is composed of complete and incomplete consensus elements (426 TEs), which were generated using REPET (Flutre et al., 2011), with a majority of TEs belonging to DNA (Class II) and less than 10% composed of retroelements (Class I). The TREP database started as a Triticeae database, but currently it includes a vast variety of elements (4162 TEs), as well as plant and non‐plant species. The top five included species in this database are *Hordeum vulgare* L., *T. aestivum, Blumeria graminis* (DC.) Speer, *O. sativa*, and *Brachypodium distachyon* (L.) P. Beauv. The Repbase v.20.05 was the largest library, with 38,777 TEs.

Phylogenetic closeness and representation of taxonomic levels of the curated library was an important factor to identify TEs. In the case of *A. thaliana*, NGSEP‐TF and RepeatMasker could annotate its genome using the curated library from the same species and Repbase. However, the performance was limited when using distant databases, such as the *C. humblotiana* database. The *A. thaliana* experiment using the species‐specific library was repeated with one to three iterations of NGSEP‐TF. As was seen with the LTR retroelements annotation, the sensitivity increased with the number of iterations (0.59 to 0.80) and the precision decreased (0.94 to 0.59) (Appendix S5). The F‐score was maximized, in this case, using two iterations (0.77).

**Figure 5:**
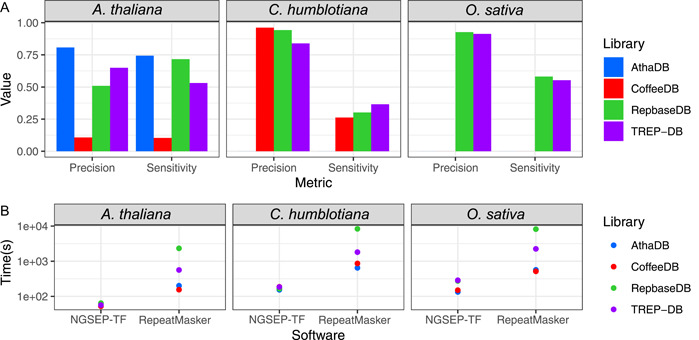
Comparison of NGSEP‐TF and RepeatMasker TE annotation for *Arabidopsis thaliana, Coffea humblotiana*, and *Oryza sativa* using the curated libraries, showing (A) the precision and sensitivity of NGSEP‐TF using RepeatMasker as a reference and (B) the time consumption of both tools using 32 cores and 64 GB of RAM.

A similar performance was observed in the *O. sativa* and *C. humblotiana* annotations. Both Repbase and the TREP database were capable of annotating the *O. sativa* genome, whereas the curated *C. humblotiana* database was useful only for the *C. humblotiana* genome. Figure 5A shows the overall precision and sensitivity of NGSEP‐TF, again using RepeatMasker as the gold standard. Although in all these cases the precision and sensitivity were not as good as using Inpactor2 libraries for LTRs, NGSEP‐TF performed well using libraries that include taxonomically close annotations. The best performance (precision and sensitivity) was achieved for *A. thaliana* when the curated library of the same species was used as input. Conversely, the best performance for *O. sativa* was achieved using Repbase, which yields results close to those obtained with the TREP database, both databases containing *O. sativa* elements. In the case of *C. humblotiana*, the precision was close to 0.96, but the sensitivity was only 0.26. A detailed investigation of the elements that could not be annotated indicates that the combination of the *k*‐mer subsampling performed in the calculation of minimizers and the reduced common *k*‐mers between the database sequences and the TEs in the *C. humblotiana* genome did not allow NGSEP‐TF to identify most elements in only two rounds. We repeated this experiment varying the number of rounds from one to 10 and found that the recall could be increased up to 0.9 at the expense of reduced precision (Appendix S6). In this case, the F‐score was maximized with five iterations, obtaining a recall of 0.74 and a precision of 0.71.

Regarding efficiency, the NGSEP‐TF annotation using a full database of TEs was at least three times faster than RepeatMasker using species‐specific libraries, and more than 20 times faster using Repbase or the TREP database (Appendices S6 and S7). The average annotation time for NGSEP‐TF was 3 min, whereas RepeatMasker required 8 min using species‐specific databases and 1 h using general databases. This is consistent with the execution time needed to annotate LTRs.

### An annotation of transposable elements for the hexaploid *Triticum aestivum* genome

The impressive reduction in computing time achieved by our algorithm allowed us to mask TEs from the hexaploid *T. aestivum* genome (15 Gbp genome size and 85% TEs) in less than 2 h using approximately 256 GB of RAM and 32 cores per chromosome. A total of 12.07 Gbp were annotated as TEs with NGSEP‐TF, corresponding to 82.83% of the genome. This amount of elements is consistent with previous studies (Zhu et al., 2021). The size distribution of TEs per chromosome was consistent among the seven chromosomes and three subgenomes, showing peaks of the distribution around 2 kbp, 8 kbp, 10 kbp, and 14 kbp (Appendix S8). Furthermore, as observed with LTR retroelements, the largest annotations corresponded to overlapped elements and TE‐rich regions. Specificity and precision of TEs within the *T. aestivum* genome could not be calculated due to the high computational resources and time that RepeatMasker requires.

**Figure 6:**
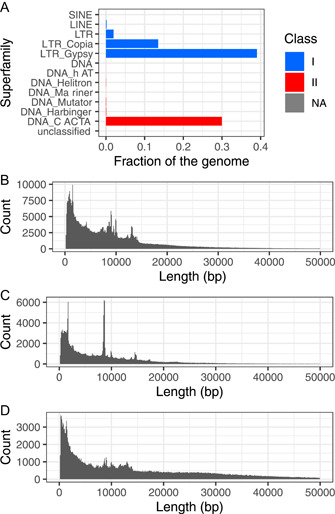
TE composition of the hexaploid *Triticum aestivum* genome. (A) Percentage of superfamilies within the genome. (B) Distribution of LTR Gypsy retrotransposons length. (C) Distribution of LTR Copia retrotransposons length. (D) Distribution of DNA CACTA transposons length.

Consistent with previous works (Zhu et al., 2021), LTR retrotransposons were the most abundant TE superfamily identified (53% of the genome), whereas the majority of LTR retroelements belong to the Gypsy (39%) and the Copia (13.5%) LTR families. The DNA CACTA superfamily was the second most abundant superfamily, corresponding to 29.9% of the genome. Figure 6A and Appendix S9 summarize the counts, total length, and percentage of all superfamilies. For the size distribution of the elements, we observed peaks of the distribution at 4 kbp, 7.5 kbp, 8 kbp, 10 kbp, and 13.5 kbp for LTR Gypsy elements, corresponding to complete elements, and some peaks below 3 kbp, composed of incomplete elements (Figure 6B). LTR Copia had a similar distribution, with the highest peak around 8 kbp, followed by a peak around 14 kbp. These peaks correspond to complete elements (Figure 6C), while peaks shorter than 4 kbp correspond to incomplete elements. CACTA elements had a flatter distribution due to their structural diversity, including a peak of small elements that reflects the presence of partial non‐autonomous elements (Figure 6D).

## CONCLUSIONS

TEs are present in a large percentage of numerous plant genomes, ranging from 3% of *Utricularia gibba* L. to over 85% of the *Zea mays* L. (maize) genome (Lee and Kim, 2014). There is a positive correlation between the percentage of TEs and the size of the genome, indicating that the largest genomes would be composed mostly of repeats (Michael, 2014). Diverging from earlier theories stating that TEs should be considered as “junk DNA” (Biémont, 2010), more recent studies show that TEs can be important drivers of genome evolution (Bourque et al., 2018), with TE dynamics producing rapid changes in chromosome organization and genome size. For example, the amplification of three LTR retrotransposon families in *Oryza australiensis* Domin (a wild relative of *O. sativa*) is involved in the dramatic genome expansion of this species (Piegu et al., 2006). TE dynamics is also an efficient mechanism to obtain novel genetic variability, which, in some cases, produces rapid innovations in phenotypic variability (Vitte et al., 2014). Despite the importance of TEs, most current genome assembly projects overlook TE annotation and therefore do not take into account the information TEs can provide on genome evolution and traits of interest. TE annotations are usually generated as a mandatory step prior to gene annotation. Consequently, TEs can be reported with variable quality and accuracy of annotations, depending on the genomes annotated. We believe that one of the main reasons for the lag in the identification and analysis of TEs in current genome assemblies is the lack of efficient and easy‐to‐use tools to perform annotation of TEs.

Most of the approaches used to discover TEs consist of creating or using a curated library and comparing it with the genome to annotate. RepeatMasker is the reference tool to perform this task, and it is included in many TE discovery pipelines, such as EDTA (Ou et al., 2019), REPET (Flutre et al., 2011), PiRATE (Berthelier et al., 2018), and TransposonUltimate (Riehl et al., 2022). However, the use of RepeatMasker requires a large server and a significant amount of time, especially for large genomes such as *T. aestivum* (15 Gbp genome size). Most other tools able to perform this task are also relatively slow to operate, in part because their core process consists of a postprocessing of computationally expensive BLAST queries. In addition to being computationally inefficient, most other available tools are also difficult to install and operate because they rely on the installation of several dependencies.

In this work, we provide an efficient tool to identify and mask most TEs present in genome assemblies. We focused our efforts on achieving a comprehensive homology‐based annotation of TEs, based on an initial database of curated elements. Inspired by algorithms that have recently been implemented to perform mapping of noisy long reads, we adapted our minimizer‐based read aligner to map the input TE sequences to the genome, which allowed us to avoid the use of BLAST or other generic sequence alignment tools. To achieve high computational efficiency, we completely bypassed the sequence alignment step and instead inferred the genomic boundaries from the cluster of minimizer hits. The described approach reduced runtime by a factor of three to 20, depending on the sizes of the input database and genome, in comparison to RepeatMasker. This reduction in time and computational requirements can represent days or even weeks for large genomes and can make it possible to perform TE annotation using small servers. Furthermore, our results are competitive when a taxonomically close database is available or is built with de novo tools, such as Inpactor2 (Orozco‐Arias et al., 2022).

Due to the *k*‐mer similarity required for the identification of shared *k*‐mers, NGSEP‐TF is more precise and accurate when input libraries include a wide representation of TEs. Our experiments indicate that the number of search iterations of the algorithm should be increased if consensus sequences are used as libraries. We recommend that users evaluate the trend of changes in the proportion of the annotated genome by increasing the number of runs. One possible limitation of using *k*‐mer hits instead of full alignments to annotate TEs is that the annotations can miss base pairs at the ends of the elements. Although our experiments suggest that the current algorithm provides adequate coverage of the annotated TEs in most cases, we will work on computationally efficient alternatives to annotate complete TEs. Complete annotation is important for downstream analysis of TEs, including assessments of diversity and divergence times. We will also explore alternatives such as hidden Markov models to increase sensitivity and completeness of the annotations and to improve the evaluation of structure and signature domains. Finally, our approach allows the annotation of longer elements than RepeatMasker due to the filtering of elements shorter than 200 bp, as they can be annotation errors (Ou et al., 2019), as well as the merging of nested annotations into larger elements. We will focus on differentiating nested elements, as they can contain fragmented or short TEs.

In addition to addressing the efficiency issue, we implemented our algorithm as part of the NGSEP to take advantage of the ease of installation and operation of this tool, as NGSEP provides intuitive command‐line and graphical user interfaces. NGSEP can be used in tandem with other recently developed tools to assemble genomes, identify TEs, align reads, identify and genotype genomic variants, and remove or select variants present within TEs. We expect that this new functionality of NGSEP to identify TEs presented here will be useful for researchers performing large‐scale genomics and genome evolution experiments across the plant kingdom.

## AUTHOR CONTRIBUTIONS

J.D. conceived and supervised the project. L.N.G.G., D.L., and J.D. developed software. L.N.G.G., J.P.L., and R.G. performed benchmark experiments. R.G. provided scientific guidance. L.N.G.G., D.L., R.G., and J.D. drafted the manuscript. All authors reviewed and approved the final version of the manuscript.

## Supporting information


**Appendix S1**. Time consumption, precision, and sensitivity when modifying the number of NGSEP‐TF iterations for LTR annotations.

**Appendix S2**. Time consumption of NGSEP‐TF and RepeatMasker for LTR annotations.

**Appendix S3**. Extended comparison of LTR retrotransposon length distribution between NGSEP‐TF and RepeatMasker for (A) *Arabidopsis thaliana*, (B) *Coffea humblotiana*, and (C) *Oryza sativa*. On the *x*‐axis, each bar represents a step of 200 bp. On the *y*‐axis, the count represents the number of LTR retrotransposons with the specified length.

**Appendix S4**. Coverage of de novo Inpactor2 TEs by NGSEP‐TF (blue) and RepeatMasker (red) annotations for (A, B) *Arabidopsis thaliana*, (C, D) *Oryza sativa*, and (E, F) *Coffea humblotiana*. (A, C, E) Coverage defined as the maximum overlap between the library and the annotation for each element over the length of the library element. (B, D, F) Coverage defined as the maximum overlap between the library and the annotation for each element over the length of the annotated element. On the *y*‐axis, the count represents the number of TEs with the specified length.

**Appendix S5**. Time consumption, precision, and sensitivity when modifying the number of NGSEP‐TF iterations for full TE annotations.

**Appendix S6**. Precision and recall of experiments to identify TEs in the *Coffea humblotiana* genome with NGSEP‐TF, using a library of consensus TEs and changing the number of rounds from one to 10. Numbers close to the data points indicate the F‐score of each data point.

**Appendix S7**. Time consumption of NGSEP‐TF and RepeatMasker for full TE annotations.

**Appendix S8**. Extended distribution of TEs across the hexaploid *Triticum aestivum* genome. Elements were annotated using NGSEP‐TF. On the *y*‐axis, the count represents the number of TEs with the specified length.

**Appendix S9**. TE composition of the hexaploid *Triticum aestivum*.

## Data Availability

The data sets supporting this article are publicly available as follows: the *Arabidopsis thaliana* TAIR10.1 genome was retrieved from the National Center for Biotechnology Information (NCBI RefSeq GCF_000001735.4); the *Oryza sativa* ‘Nipponbare’ genome assembly was downloaded from the website of the Rice Genome Annotation Project (http://rice.uga.edu/); the *Coffea humblotiana* genome was retrieved from the Sol Genomics Network (2023); and the hexaploid genome of *Triticum aestivum* was retrieved from NCBI RefSeq (GCA_018294505.1). The *C. humblotiana* curated TE library is available at https://solgenomics.net/ftp/genomes/Coffea_humblotiana/hum_refTEs.fa.txt (Sol Genomics Network, 2023); the *A. thaliana* library was downloaded from https://urgi.versailles.inra.fr/Data/Transposable-elements/Arabidopsis (Plant Bioinformatics Facility, 2023); and the TREP database is available at https://trep-db.uzh.ch (Schlagenhauf and Wicker, 2016). NGSEP is distributed without restriction as an open‐source Java software package under the GNU General Public License v3. The source code of every released version of NGSEP, including the version used in this study, is available at the NGSEP SourceForge web page (https://sourceforge.net/projects/ngsep/).
