# Supplementary material for: Efficient homology‐based annotation of transposable elements using minimizers
Source: Appl Plant Sci. 2023 May 11;11(4):e11520. doi: 10.1002/aps3.11520 (PMC10439823; doi:10.1002/aps3.11520)
Supplement: Supplementary file 1 — Appendix S1. Time consumption, precision, and sensitivity when modifying the number of NGSEP‐TF iterations for LTR annotations. [file APS3-11-e11520-s005.docx]

**Appendix S1.** Time consumption, precision, and sensitivity when modifying the number of NGSEP-TF iterations for LTR annotations.

| **Library** | **Species** | **Precision** | **Sensitivity** | **F-score** | **Iterations** | **Time (s)** |
| --- | --- | --- | --- | --- | --- | --- |
| Inpactor2 de novo Library | *Arabidopsis thaliana* | 0.9608 | 0.6596 | 0.782 | 1 | 18.98 |
| Inpactor2 de novo Library | *A. thaliana* | 0.7508 | 0.7841 | 0.767 | 2 | 19.99 |
| Inpactor2 de novo Library | *A. thaliana* | 0.4781 | 0.8362 | 0.608 | 3 | 23.49 |
| Inpactor2 de novo Library | *Oryza sativa* | 0.9857 | 0.7016 | 0.819 | 1 | 55.28 |
| Inpactor2 de novo Library | *O. sativa* | 0.8155 | 0.8315 | 0.823 | 2 | 140.10 |
| Inpactor2 de novo Library | *O. sativa* | 0.5697 | 0.8938 | 0.695 | 3 | 301.08 |
