# Supplementary material for: Efficient homology‐based annotation of transposable elements using minimizers
Source: Appl Plant Sci. 2023 May 11;11(4):e11520. doi: 10.1002/aps3.11520 (PMC10439823; doi:10.1002/aps3.11520)
Supplement: Supplementary file 2 — Appendix S2. Time consumption of NGSEP‐TF and RepeatMasker for LTR annotations. [file APS3-11-e11520-s008.docx]

**Appendix S2.** Time consumption of NGSEP-TF and RepeatMasker for LTR annotations.

| **Species** | **Library** | **NGSEP-TF (s)** | **RepeatMasker (s)** | **Factor** |
| --- | --- | --- | --- | --- |
| *Arabidopsis thaliana* | Inpactor2 de novo Library | 57.0 | 169.0 | 296.58% |
| *Coffea humblotiana* | Inpactor2 de novo Library | 261.2 | 3529.0 | 1350.93% |
| *Oryza sativa* | Inpactor2 de novo Library | 357.5 | 1805.0 | 504.90% |
| *A. thaliana* | non-redundant InpactorDB | 131.0 | 12577.0 | 9600.10% |
| *C. humblotiana* | non-redundant InpactorDB | 254.4 | 46979.0 | 18465.50% |
| *O. sativa* | non-redundant InpactorDB | 481.9 | 44425.0 | 9217.80% |
| *A. thaliana* | redundant InpactorDB | 200.2 | 24233.0 | 12102.88% |
| *C. humblotiana* | redundant InpactorDB | 342.6 | 89639.0 | 26164.41% |
| *O. sativa* | redundant InpactorDB | 653.1 | 96210.0 | 14730.38% |
| *A. thaliana* | non-redundant InpactorDB + Inpactor2 de novo library | 116.0 | 11422.0 | 9845.62% |
| *C. humblotiana* | non-redundant InpactorDB + Inpactor2 de novo library | 319.5 | 50531.0 | 15816.94% |
| *O. sativa* | non-redundant InpactorDB + Inpactor2 de novo library | 494.4 | 51208.0 | 10357.84% |
