# Supplementary material for: Efficient homology‐based annotation of transposable elements using minimizers
Source: Appl Plant Sci. 2023 May 11;11(4):e11520. doi: 10.1002/aps3.11520 (PMC10439823; doi:10.1002/aps3.11520)

DOI 10.1002/aps3.11520

APPENDIX S3. Extended comparison of LTR retrotransposon length distribution between NGSEP-TF and RepeatMasker for (A) *Arabidopsis thaliana*, (B) *Coffea humblotiana*, and (C) *Oryza sativa*. On the *x*-axis, each bar represents a step of 200 bp. On the *y*-axis, the count represents the number of LTR retrotransposons with the specified length.

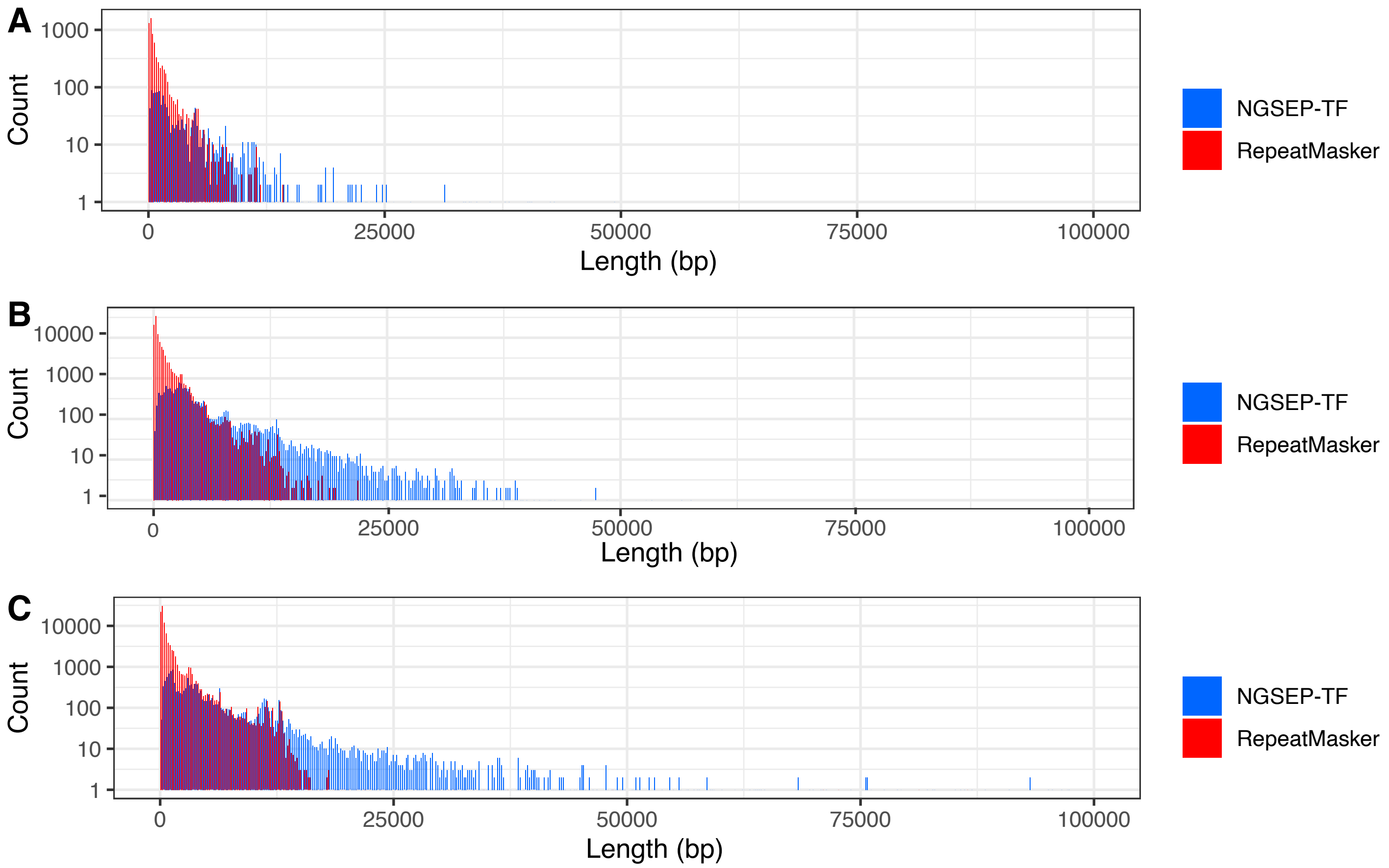

Supplement: Supplementary file 3 — Appendix S3. Extended comparison of LTR retrotransposon length distribution between NGSEP‐TF and RepeatMasker for (A) Arabidopsis thaliana, (B) Coffea humblotiana, and (C) Oryza sativa. On the x‐axis, each bar represents a step of 200 bp. On the y‐axis, the count represents the number of LTR retrotransposons with the specified length. [file APS3-11-e11520-s004.pdf]
