# Supplementary material for: Efficient homology‐based annotation of transposable elements using minimizers
Source: Appl Plant Sci. 2023 May 11;11(4):e11520. doi: 10.1002/aps3.11520 (PMC10439823; doi:10.1002/aps3.11520)

DOI 10.1002/aps3.11520

APPENDIX S4. Coverage of de novo Inpactor2 TEs by NGSEP-TF (blue) and RepeatMasker (red) annotations for (A, B) *Arabidopsis thaliana*, (C, D) *Oryza sativa*, and (E, F) *Coffea humblotiana*. (A, C, E) Coverage defined as the maximum overlap between the library and the annotation for each element over the length of the library element. (B, D, F) Coverage defined as the maximum overlap between the library and the annotation for each element over the length of the annotated element. On the *y*-axis, the count represents the number of TEs with the specified length.

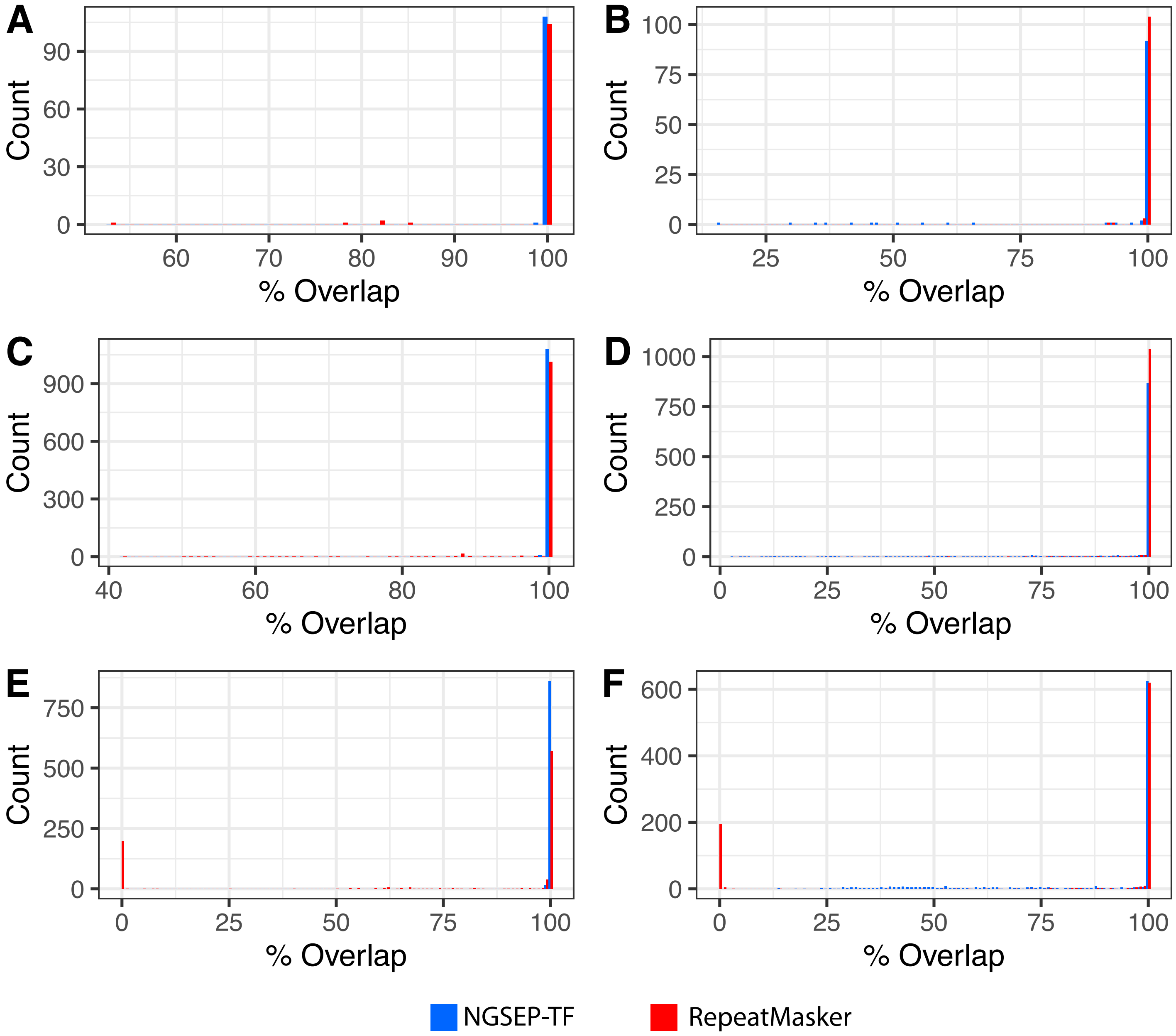

Supplement: Supplementary file 4 — Appendix S4. Coverage of de novo Inpactor2 TEs by NGSEP‐TF (blue) and RepeatMasker (red) annotations for (A, B) Arabidopsis thaliana, (C, D) Oryza sativa, and (E, F) Coffea humblotiana. (A, C, E) Coverage defined as the maximum overlap between the library and the annotation for each element over the length of the library element. (B, D, F) Coverage defined as the maximum overlap between the library and the annotation for each element over the length of the annotated element. On the y‐axis, the count represents the number of TEs with the specified length. [file APS3-11-e11520-s009.pdf]
