# Supplementary material for: Efficient homology‐based annotation of transposable elements using minimizers
Source: Appl Plant Sci. 2023 May 11;11(4):e11520. doi: 10.1002/aps3.11520 (PMC10439823; doi:10.1002/aps3.11520)
Supplement: Supplementary file 5 — Appendix S5. Time consumption, precision, and sensitivity when modifying the number of NGSEP‐TF iterations for full TE annotations. [file APS3-11-e11520-s006.docx]

**Appendix S5.** Time consumption, precision, and sensitivity when modifying the number of NGSEP-TF iterations for full TE annotations.

| **Library** | **Species** | **Precision** | **Sensitivity** | **F-score** | **Iterations** | **Time (s)** |
| --- | --- | --- | --- | --- | --- | --- |
| AthaDB | *Arabidopsis thaliana* | 0.9492 | 0.5988 | 0.734 | 1 | 20.12 |
| AthaDB | *A. thaliana* | 0.8076 | 0.7433 | 0.774 | 2 | 25.84 |
| AthaDB | *A. thaliana* | 0.5951 | 0.8066 | 0.684 | 3 | 37.22 |
