# Supplementary material for: Efficient homology‐based annotation of transposable elements using minimizers
Source: Appl Plant Sci. 2023 May 11;11(4):e11520. doi: 10.1002/aps3.11520 (PMC10439823; doi:10.1002/aps3.11520)

DOI 10.1002/aps3.11520

APPENDIX S6. Precision and recall of experiments to identify TEs in the *Coffea humblotiana* genome with NGSEP-TF, using a library of consensus TEs and changing the number of rounds from one to 10. Numbers close to the data points indicate the F-score of each data point.

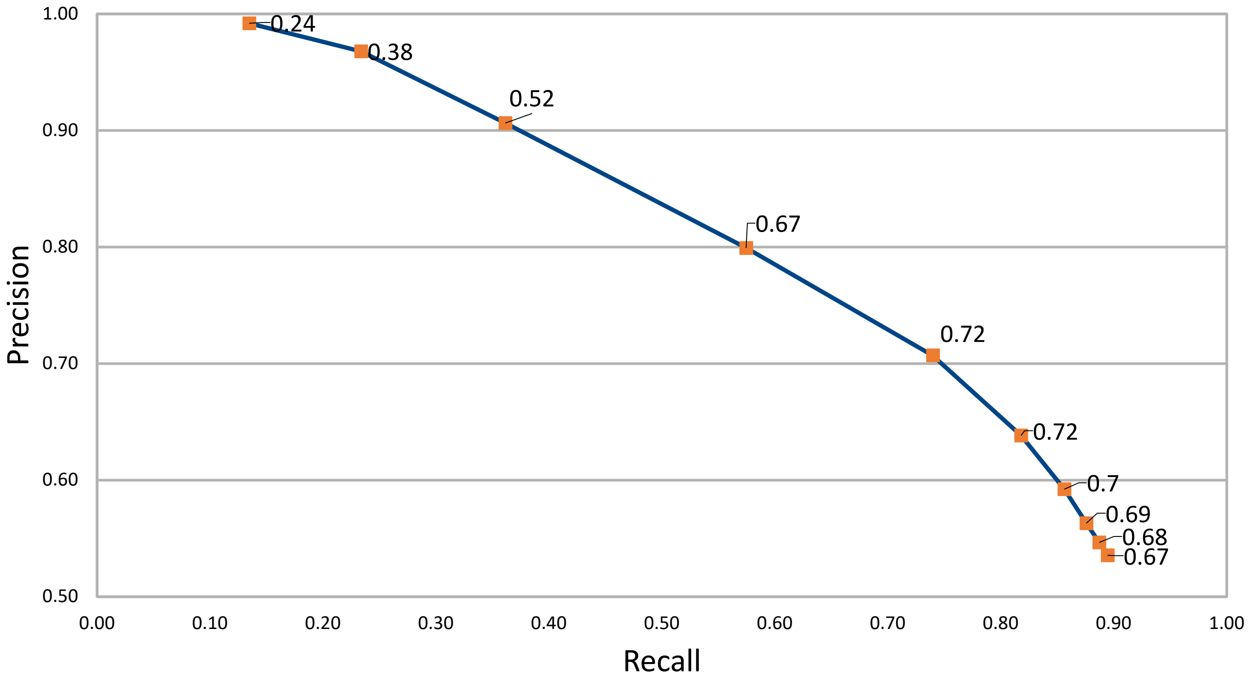

Supplement: Supplementary file 6 — Appendix S6. Precision and recall of experiments to identify TEs in the Coffea humblotiana genome with NGSEP‐TF, using a library of consensus TEs and changing the number of rounds from one to 10. Numbers close to the data points indicate the F‐score of each data point. [file APS3-11-e11520-s007.pdf]
