# Supplementary material for: Efficient homology‐based annotation of transposable elements using minimizers
Source: Appl Plant Sci. 2023 May 11;11(4):e11520. doi: 10.1002/aps3.11520 (PMC10439823; doi:10.1002/aps3.11520)
Supplement: Supplementary file 7 — Appendix S7. Time consumption of NGSEP‐TF and RepeatMasker for full TE annotations. [file APS3-11-e11520-s003.docx]

**Appendix S7.** Time consumption of NGSEP-TF and RepeatMasker for full TE annotations.

| **Species** | **Library^a^** | **NGSEP-TF (s)** | **RepeatMasker (s)** | **Factor** |
| --- | --- | --- | --- | --- |
| *Arabidopsis thaliana* | AthaDB | 62.3 | 201.0 | 322.71% |
| *Coffea humblotiana* | AthaDB | 153.8 | 648.0 | 421.23% |
| *Oryza sativa* | AthaDB | 133.5 | 568.0 | 425.39% |
| *A. thaliana* | CoffeeDB | 52.3 | 155.0 | 296.37% |
| *C. humblotiana* | CoffeeDB | 187.3 | 865.0 | 461.78% |
| *O. sativa* | CoffeeDB | 149.9 | 516.0 | 344.28% |
| *A. thaliana* | Repbase | 64.1 | 2317.0 | 3615.34% |
| *C. humblotiana* | Repbase | 165.0 | 8331.0 | 5048.54% |
| *O. sativa* | Repbase | 274.97 | 8157 | 2966.51% |
| *A. thaliana* | TREP-DB | 56.4 | 564.0 | 1000.75% |
| *C. humblotiana* | TREP-DB | 185.0 | 1807.0 | 976.72% |
| *O. sativa* | TREP-DB | 287.6 | 2239.0 | 778.47% |

^a^Libraries are: AthaDB = *Arabidopsis thaliana* library (<https://urgi.versailles.inra.fr/Data/Transposable-elements/Arabidopsis>); CoffeaDB = *Coffea humblotiana* curated TE library (<https://solgenomics.net/ftp/genomes/Coffea_humblotiana/hum_refTEs.fa.txt>); Repbase library v.20.05 (Bao et al., 2015); TREP-DB = TREP database ([https://trep-db.uzh.ch](https://trep-db.uzh.ch/)).

**REFERENCES**

Bao, W., K. K. Kojima, and O. Kohany. 2015. Repbase Update, a database of repetitive elements in eukaryotic genomes. *Mobile DNA* 6: 11. https://doi.org/10.1186/s13100-015-0041-9
