# Supplementary material for: Efficient homology‐based annotation of transposable elements using minimizers
Source: Appl Plant Sci. 2023 May 11;11(4):e11520. doi: 10.1002/aps3.11520 (PMC10439823; doi:10.1002/aps3.11520)

DOI 10.1002/aps3.11520

APPENDIX S8. Extended distribution of TEs across the hexaploid *Triticum aestivum* genome. Elements were annotated using NGSEP-TF. On the *y*-axis, the count represents the number of TEs with the specified length.

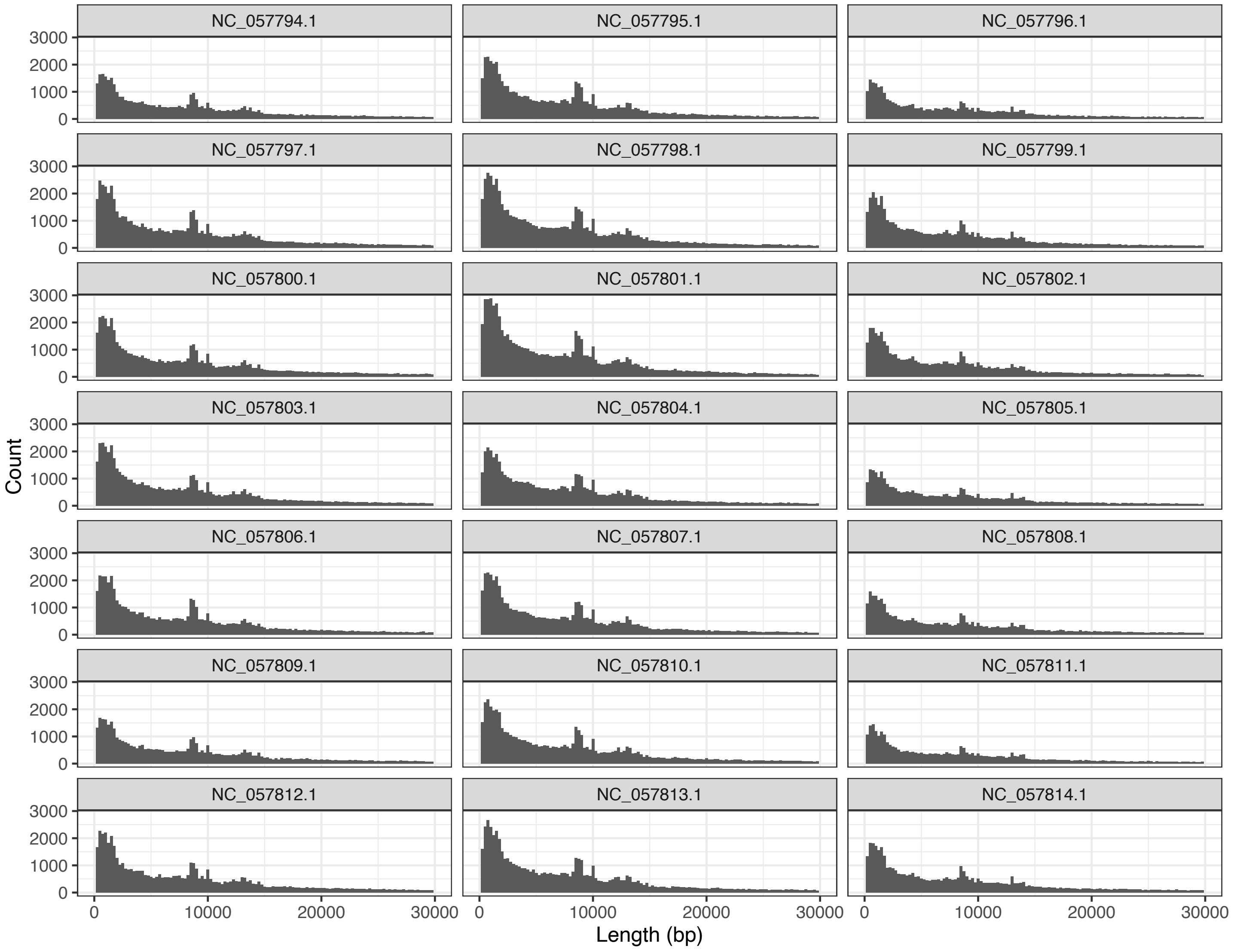

Supplement: Supplementary file 8 — Appendix S8. Extended distribution of TEs across the hexaploid Triticum aestivum genome. Elements were annotated using NGSEP‐TF. On the y‐axis, the count represents the number of TEs with the specified length. [file APS3-11-e11520-s002.pdf]
