# Supplementary material for: Efficient homology‐based annotation of transposable elements using minimizers
Source: Appl Plant Sci. 2023 May 11;11(4):e11520. doi: 10.1002/aps3.11520 (PMC10439823; doi:10.1002/aps3.11520)
Supplement: Supplementary file 9 — Appendix S9. TE composition of the hexaploid Triticum aestivum. [file APS3-11-e11520-s001.docx]

**Appendix S9.** TE composition of the hexaploid *Triticum aestivum*.

| **Superfamily*** | **Abbreviation** | **Class** | **Count** | **Percentage** | **Total length** | **Fraction of the genome** |
| --- | --- | --- | --- | --- | --- | --- |
| LTR_Copia | RLC | I | 219,483 | 18.98% | 1,919,237,035 | 13.49% |
| LTR_Gypsy | RLG | I | 585,223 | 50.63% | 5,555,623,866 | 39.05% |
| LTR | RLX | I | 37,837 | 3.30% | 279,315,679 | 1.96% |
| LINE | RIX/RII | I | 9653 | 0.83% | 23,043,540 | 0.16% |
| SINE | RSX | I | 3592 | 0.31% | 1,915,393 | 0.01% |
| DNA_Helitron | DHH | II | 1444 | 0.12% | 4,964,556 | 0.03% |
| DNA_hAT | DTA | II | 100 | 0.00% | 90,210 | 0.00% |
| DNA_CACTA | DTC | II | 272,957 | 23.61% | 4,264,394,182 | 29.98% |
| DNA_Harbinger | DTH | II | 11,676 | 1.01% | 12,017,004 | 0.08% |
| DNA_Mutator | DTM | II | 7469 | 0.64% | 8,000,196 | 0.06% |
| DNA_Mariner | DTT | II | 1694 | 0.14% | 472,263 | 0.00% |
| DNA | DTX/DXX | II | 1154 | 0.09% | 473,078 | 0.00% |
| Unclassified | XXX | NA | 3618 | 0.31% | 5,303,198 | 0.04% |

*Transposable element superfamilies were defined by Wicker et al. (2007).

**REFERENCES**

Wicker, T., F. Sabot, A. Hua-Van, J. L. Bennetzen, P. Capy, B. Chalhoub, A. Flavell, et al. 2007. A unified classification system for eukaryotic transposable elements. *Nature Reviews Genetics* 8: 973–982.
